# Réactivation d'hépatite virale B chez un patient traité pour lymphome non hodgkinien B diffus à grandes cellules par rituximab: à propos d'un cas

**DOI:** 10.11604/pamj.2015.22.22.7579

**Published:** 2015-09-10

**Authors:** Bienvenu Houssou, Romaric Mahutondji Massi, Marième Camara, Hassan Mifdal, Nadia Nourichafi, Saadia Zafad, Bouchra Oukkache

**Affiliations:** 1 Centre Régional de Transfusion Sanguine de Casablanca, Maroc; 2 Laboratoire d'Hématologie du CHU Ibn Rochd, Casablanca, Maroc; 3 Clinique privée, Casablanca, Maroc

**Keywords:** Hépatite virale B, immunosuppression, réactivation, hepatitis B virus, immunosuppression, reactivation

## Abstract

La réactivation du virus de l'hépatite B est secondaire à une diminution de l'immunité de l'hôte et peut être suivie d'une poussée d'hépatite aigue potentiellement mortelle. Nous rapportons le cas d'un patient D.H, 47 ans, sexe masculin, AgHBs négatif, jamais transfusé, jamais vacciné contre l'hépatite B qui avait présenté en mars 2013 un LNH B diffus à grandes cellules stade IV par moelle. Traité par 8 cures R-CHOP, il était en rémission complète clinique et paraclinique. Neuf mois après, il fait une rechute de son lymphome classé stade III, associée à une hépatite virale B en réplication virale (18.000 copies/mL): réactivation virale B chez porteur occulte traité avec entécavir 0.5mg par jour pendant 6 mois, l'ADN du VHB était indétectable en fin de traitement. Il avait reçu deux cures de DHAP puis deux cures R-DHAP avec une rémission complète. Lors du recueil des cellules souches en vue de l'autogreffe, l'AgHBs est à nouveau positif. Il a été greffé le 12/01/2015 et continue son traitement antiviral pour 6 mois encore.

## Introduction

La réactivation du virus de l'hépatite B (VHB) est un syndrome bien défini caractérisé par l'augmentation de la virémie VHB; habituellement de plus de 1 log10 UI/ml chez un patient porteur occulte du VHB ou connu pour une infection VHB chronique ou même guérie [1]. Cette réactivation virale est en générale secondaire à une diminution de l'immunité de l'hôte et peut être suivie d'une poussée d'hépatite aigue potentiellement mortelle. Les principaux facteurs de risque de réactivation incriminés sont: les anticorps monoclonaux comme le rituximab, les corticoïdes, et chimiothérapies [2]. La fréquence de la réactivation virale B au cours des chimiothérapies chez les patients porteurs chroniques du VHB avec Ag HBs positifs est en moyenne de 46%. Cette fréquence est en moyenne de 5% chez les porteurs occultes du virus B [3]. Pour les patients traités avec le rituximab, une étude prospective récente a démontré que le risque cumulatif sur deux ans de la réactivation du VHB est estimé à 41,5% [4]. Au Maroc, la prévalence de l'AgHBs est estimée actuellement à 1, 66% [5]. La prévalence du portage occulte du VHB n'est pas connue ainsi que la fréquence des réactivations du VHB au cours des chimiothérapies et ou traitement immunosuppresseur. Nous rapportons un cas de réactivation du virus de l'hépatite B suite à un traitement par le rituximab.

## Patient et observation

D.H, 47 ans, sexe masculin, avait présenté en mars 2013 un lymphome non hodgkinien diffus à grandes cellules B à localisation ganglionnaire et classé stade IV par moëlle. Il n’était pas connu porteur d'hépatite B, n'a jamais été transfusé et n'a pas été vacciné contre l'hépatite B. Dans le cadre du bilan pré-thérapeutique: l'AgHBs, l'anticorps anti HCV et la sérologie HIV étaient négatifs. Il a été mis sous protocole R-CHOP (rituximab, oncovin, endoxan, adriamycine, prednisone). Une rémission complète clinique et paraclinique a été obtenue au terme des 8 cures.

Neuf mois après, le patient avait présenté une rechute de son lymphome classé stade III. La biopsie ostéomédullaire était normale. La sérologie de l'hépatite B était positive complétée avec PCR qui objectivait une réplication virale B à 18.000 copies/mL. Les transaminases étaient élevées: ASAT à 402 UI/L (Normale inférieure à 40UI/L), ALAT à 384UI/L (Normale inférieure à 42UI/L). Une discussion multidisciplinaire sur le dossier a été tenue entre hématologues, gastroentérologues et infectiologues et l'hypothèse d'une réactivation virale B chez porteur occulte a été émise. Il a été décidé de: démarrer le traitement antiviral B avec antécavir 0.5mg/jour pour une durée de six mois; démarrer le traitement de rattrapage du lymphome non hodgkinien avec chimiothérapie type DHAP (cisplatine, aracytine, dexaméthasone, chibrocadron); introduire le rituximab dès négativation de la charge virale suivie d'une autogreffe.

L’évolution clinique, biochimique et virologique était favorable. Le patient était asymptomatique avec un taux de transaminases normal; la quantification de l'ADN du VHB était indétectable (moins 200 copies/mL) après deux cures de DHAP. La troisième et la quatrième cure ont été administrées avec du rituximab. Reçu le 20 /11/2014 au Centre Régional de transfusion Sanguine de Casablanca pour le prélèvement de cellules souches où un bilan sérologique avait montré une antigénémie Ag HBs positive. Le diagnostic de réactivation virale B en rapport avec l'administration du rituximab a été retenu. Le prélèvement de cellules souches a été réalisé le 25 /11/2014. Le patient a été greffé le 12/01/2015 et continue son traitement antiviral pour 6 mois encore.

## Discussion

La réactivation virale B peut être asymptomatique comme elle peut engendrer des lésions graves engageant le pronostic vital même en présence d'un traitement antiviral. En engendrant une immunosuppression, la chimiothérapie va faciliter la réplication virale. La restauration de l’état immunitaire survenant durant les périodes inter cycles ou quelques semaines à quelques mois après la fin de la chimiothérapie, peut causer des lésions hépatiques importantes donc une hépatite aigue fulminante responsable des décès dans les réactivations virales B [6]. Les réactivations modérées peuvent être vues sans retentissement clinique.

Une revue systématique récente, incluant quatorze études et 475 patients AgHBs positifs ayant une chimiothérapie sans prophylaxie VHB, a démontré un taux de réactivation compris entre 24% et 88%; et une mortalité liée à la réactivation VHB de 7% [3]. Dans une étude ayant suivi de façon longitudinale 244 patients asiatiques Ag HBs négatifs avant le début de la chimiothérapie, mais dont 61% avaient été en contact avec le virus B, une réactivation est apparue dans 8 cas (3,3%) [7]. Le rituximab, un anticorps monoclonal anti-CD20 utilisé en onco-hématologie, est à l'origine d'un risque élevé de réactivation virale B. Une étude rétrospective de 115 patients, traités pour un lymphome avec des régimes à base de rituximab, a démontré un taux de poussées du VHB de 80% chez les dix patients AgHBs positifs, dont un décès [8]. De plus, 4,2% des patients AgHBs négatifs ont également développé des poussées de VHB, dont deux menant à des décès (2,1% des patients AgHBs négatifs). Un autre facteur de risque majeur pour la réactivation VHB est la transplantation allogénique de moelle. Chez les patients AgHBs positifs, la réactivation virale VHB est quasiment universelle [9].

Notre observation, illustre un cas de réactivation virale B chez un porteur occulte et met en exergue que la pratique quotidienne consistant à ne demander que l'AgHBs pour le dépistage d'une éventuelle infection au VHB avant une chimiothérapie et ou un traitement immunosuppresseur est insuffisante. La Société Européenne d'Hépatologie (European Association for the Study of the Liver, EASL) recommande un dépistage du VHB à tous les patients candidats à une chimiothérapie ou une immunosuppression, y compris par des agents biologiques [10]. Le dépistage devrait comporter au moins l'antigène HBs et les anticorps anti-HBs et anti-HBc. La positivité de l'anticorps anti-HBc chez un sujet non vacciné doit faire évoquer un portage occulte du VHB. Dans tous les cas, si un de ces marqueurs est positif, un dépistage sérologique du VHB complet devrait être effectué: ADN VHB par RT-PCR, Ag HBe, Ac anti-HBe et, selon la situation clinique, un dépistage de l'hépatite D. -Si le dépistage du VHB est négatif, une vaccination contre ce virus est fortement recommandée [10]. Si le dépistage est positif: AgHBs +, l'attitude est fonction de la charge virale détectée par RT-PCR. Lorsqu'elle est inférieure à 2000 copies/ml, traitement préventif systématique par la Lamivudine pendant la chimiothérapie et/ou l'immunosuppression et douze mois après. Lorsqu'elle est supérieure à 2000 copies/ml, traitement préventif systématique par l'entécavir ou le ténofovir pendant la chimiothérapie et/ou l'immunosuppression et douze mois après. Si le dépistage objective un portage occulte: AgHBs- et Ac anti-HBc +, l'attitude est fonction de la détection ou non du virus par RT-PCR. Lorsque le virus est détecté par RT-PCR, la prise en charge est identique à celle du dépistage positif. Lorsque la RT-PCR ne détecte pas de charge virale: Ac HBs - ou Ac HBs +, on fait soit une surveillance de la charge virale soit le traitement systématiquement avec de la lamivudine.

De multiples études ont mis en évidence le bénéfice à introduire un traitement préventif chez les patients à risque d'une réactivation du VHB dans le contexte d'une immunosuppression. Une revue systématique a démontré le bénéfice de la lamivudine, un analogue nucléosidique, dans ce contexte. Cette revue a inclu quatorze études (deux études randomisées contrôlées, huit cohortes prospectives et quatre rétrospectives) avec 275 patients dans le bras lamivudine et 475 patients « contrôle ». Aucun patient dans le groupe lamivudine n'a développé une insuffisance hépatique liée au VHB, contre 13% des patients du groupe contrôle. Par ailleurs, la mortalité attribuée au VHB était de 2% dans le groupe lamivudine mais de 7% dans le groupe contrôle. Aucun effet secondaire important de la lamivudine n'a été rapporté dans cette revue. Ainsi, le traitement prophylactique du VHB par lamivudine dans un contexte d'immunosuppression est efficace, sûr et simple à administrer (un comprimé quotidien) [3].

## Conclusion

La réactivation virale B dans un contexte d'immunosuppression peut se révéler fatale pour le patient. Toute introduction de médicaments immunosuppresseurs doit faire rechercher obligatoirement une potentielle infection par le VHB (au minimum, AgHBs, Ac anti HBc). En cas de dépistage positif un traitement prophylactique devra être institué.
